# Necessity and Content of Swing Phase Gait Coordination Training Post Stroke; A Case Report

**DOI:** 10.3390/brainsci11111498

**Published:** 2021-11-12

**Authors:** Jessica P. McCabe, Kristen Roenigk, Janis J. Daly

**Affiliations:** 1 Cognitive and Motor Learning Program, Cleveland VA Medical Center, Cleveland, OH 44106, USA; kristen.roenigk@gmail.com; 2 Brain Rehabilitation Research Center, Malcom Randall VA Medical Center, Gainesville, FL 32608, USA; 3 College of Public Health and Health Professions, University of Florida, Gainesville, FL 32608, USA

**Keywords:** stroke, gait, coordination, motor learning, body weight supported treadmill training, functional electrical stimulation (FES), mobility, life role participation

## Abstract

Background/Problem: Standard neurorehabilitation and gait training has not proved effective in restoring normal gait coordination for many stroke survivors. Rather, persistent gait dyscoordination occurs, with associated poor function, and progressively deteriorating quality of life. One difficulty is the array of symptoms exhibited by stroke survivors with gait deficits. Some researchers have addressed lower limb weakness following stroke with exercises designed to strengthen muscles, with the expectation of improving gait. However, gait dyscoordination in many stroke survivors appears to result from more than straightforward muscle weakness. Purpose: Thus, the purpose of this case study is to report results of long-duration gait coordination training in an individual with initial good strength, but poor gait swing phase hip/knee and ankle coordination. Methods: Mr. X was enrolled at >6 months after a left hemisphere ischemic stroke. Gait deficits included a ‘stiff-legged gait’ characterized by the absence of hip and knee flexion during right mid-swing, despite the fact that he showed good initial strength in right lower limb quadriceps, hamstrings, and ankle dorsiflexors. Treatment was provided 4 times/week for 1.5 h, for 12 weeks. The combined treatment included the following: motor learning exercises designed for coordination training of the lower limb; functional electrical stimulation (FES) assisted practice; weight-supported coordination practice; and over-ground and treadmill walking. The FES was used as an adjunct to enhance muscle response during motor learning and prior to volitional recovery of motor control. Weight-supported treadmill training was administered to titrate weight and pressure applied at the joints and to the plantar foot surface during stance phase and pre-swing phase of the involved limb. Later in the protocol, treadmill training was administered to improve speed of movement during the gait cycle. Response to treatment was assessed through an array of impairment, functional mobility, and life role participation measures. Results: At post-treatment, Mr. X exhibited some recovery of hip, knee, and ankle coordination during swing phase according to kinematic measures, and the stiff-legged gait was resolved. Muscle strength measures remained essentially constant throughout the study. The modified Ashworth scale showed improved knee extensor tone from baseline of 1 to normal (0) at post-treatment. Gait coordination overall improved by 12 points according to the Gait Assessment and Intervention Tool, Six Minute Walk Test improved by 532′, and the Stroke Impact Scale improved by 12 points, including changes in daily activities; mobility; and meaningful activities. Discussion: Through the combined use of motor learning exercises, FES, weight-support, and treadmill training, coordination of the right lower limb improved sufficiently to exhibit a more normal swing phase, reducing the probability of falls, and subsequent downwardly spiraling dysfunction. The recovery of lower limb coordination during swing phase illustrates what is possible when strength is sufficient and when coordination training is targeted in a carefully titrated, highly incrementalized manner. Conclusions/Contribution to the Field: This case study contributes to the literature in several ways: (1) illustrates combined interventions for gait training and response to treatment; (2) provides supporting case evidence of relationships among knee flexion coordination, swing phase coordination, functional mobility, and quality of life; (3) illustrates that strength is necessary, but not sufficient to restore coordinated gait swing phase after stroke in some stroke survivors; and (4) provides details regarding coordination training and progression of gait training treatment for stroke survivors.

## 1. Introduction

Standard neurorehabilitation and gait training has not proved effective in restoring normal gait coordination for many stroke survivors. Rather, persistent gait dyscoordination occurs, with associated poor function, and progressively deteriorating quality of life [1].

One difficulty is the array of symptoms exhibited by stroke survivors with gait deficits. Some researchers have addressed lower limb weakness following stroke with exercises designed to strengthen muscles, with the expectation of improving gait. However, gait dyscoordination [2] in many stroke survivors appears to result from more than straightforward muscle weakness. Dyscoordination is a treatment-resistant, though highly relevant, impairment underlying some gait deficits after stroke. This dyscoordination can be influenced by one or more of the following: spasticity [3,4]¸ sensory loss [5], balance impairment [6], and inability to selectively activate a muscle in an isolated manner [7,8,9,10]; that is, abnormal co-contraction of an antagonistic muscle occurs, which is counter to the desired action [7,8,9,10]. Therefore, there is a need for comprehensive treatment approaches that address gait dyscoordination and the array of contributing factors.

One commonly observed, treatment-resistant gait coordination deficit is insufficient or absent hip and knee flexion in swing phase, sometimes clinically termed “stiff-legged gait” [11,12,13]. In this case, individuals are unable to normally flex the hip and/or knee during the swing phase, which results in compensatory strategies. These clinically familiar compensatory strategies can include one or more of the following: shortened swing phase in which the involved swing limb ends swing phase early by simply ‘stepping up to’ the position just next to (not beyond, as would normally occur) the opposite stance limb; circumduction of the involved limb by moving the ‘stiff’ limb’ in a rotational arc arising from the hip; excessively plantarflexing the uninvolved stance limb ankle (“vaulting”) so that the abnormally straight swinging limb can clear the floor; and laterally leaning toward the uninvolved stance limb for the straight involved limb to clear the floor during its swing phase [14]. Existing evidence of strengthening exercises has shown improvement in some gait characteristics [15,16,17,18,19,20,21], but the exercises provided in these studies did not resolve dyscoordinated gait. Therefore, to address this gap in the literature, this case study of a chronic stroke survivor illustrates response to gait coordination training in the presence of baseline normal muscle strength throughout almost all lower limb muscles at baseline of the study, but also baseline swing phase, treatment-resistant gait dyscoordination.

## 2. Methods

### 2.1. Case Characteristics

Mr. X was age 67, having had a left hemisphere, basal ganglion stroke 6 years prior to enrollment in the study. The participant gave his written informed consent for inclusion before he participated in the study. The study was conducted in accordance with the Declaration of Helsinki, and the protocol was approved by the Ethics Committee of the Louis Stokes VA Medical Center (Internal Review Board (IRB; Project identification code, CPA#922)). 

### 2.2. Measures

Assessments were conducted by a blinded examiner at baseline and at the end of 3 months of treatment. Impairment measures included the following: lower limb coordination using the Fugl-Meyer-Lower Extremity scale (FM) [22]; muscle strength, standard manual muscle testing (MMT) on a 5-point scale [23]; mAshworth muscle tone tested in a static position [24]; gait coordination according to the Gait Assessment and Intervention Tool (G.A.I.T) [25,26,27]; and kinematics of the gait events at mid-swing phase for hip, knee, and ankle flexion, using standard motion capture system acquisition methods (Vicon 370; Oxford Metrics, UK; a three-dimensional video data acquisition system on a 30 foot walkway, with seven device cameras and 15 reflective markers at pelvic and limb landmarks (modified Hayes configuration)). We averaged kinematic data across 30 strides of chosen speed by calculating limb position coordinates and conducting three-dimensional reconstruction. Electromyographic data (J&J Engineering System (Poulsbo, WA, USA)) were acquired with acquisition parameters as follows: 1100 Hz sampling frequency (band-pass filter = 40–360 Hz with a 1 pole Chebyshev filter) with active bipolar surface electrodes (silver/silver chloride strips 1 cm square and 1 cm apart, along the longitudinal axis of the muscle). Further details are published elsewhere [28]. We identified mean muscle latencies (time between command to move and EMG signal onset) across 10 repetitions of ballistic knee flexion motor tasks in two static body positions, as follows: (1) side-lying, with the hip in a hip-neutral position throughout; and (2) standing on the left leg, with the right limb in the trailing position with toe touching the ground behind, ready to flex the knee. Muscles studied were: hamstring knee flexor muscles (short head of the biceps femoris (SH); long head of the biceps femoris (LH)); quadriceps knee extensor muscles (vastus medialis (VM) and vastus lateralis (VL)). We also acquired EMG data from right, involved limb muscles, during chosen speed walking and generated muscle activation and deactivation times according to the standard method of percent of the gait cycle (muscles studied: tibialis anterior (TA), hamstrings (LH), quadriceps (VL), and gluteus maximus (GM)).

Functional mobility measure was the 6 Minute Walk Test (6MWT), with a minimal clinically important difference (MCID) of 233 ft for gait speed of >0.4 m/s [29]. Quality of Life (or life role participation) was assessed using the Stroke Impact Scale (SIS), for which a change of 10–15 points represents a meaningful change [30].

### 2.3. Interventions

The treatment protocol was administered in sessions of 1.5 h, four times/week for 12 weeks (72 h, total) in a one-to-one therapist: participant ratio by a physical therapist with years of stroke neurorehabilitation experience.

#### 2.3.1. Motor Learning Treatment Component

**Figure 1:**
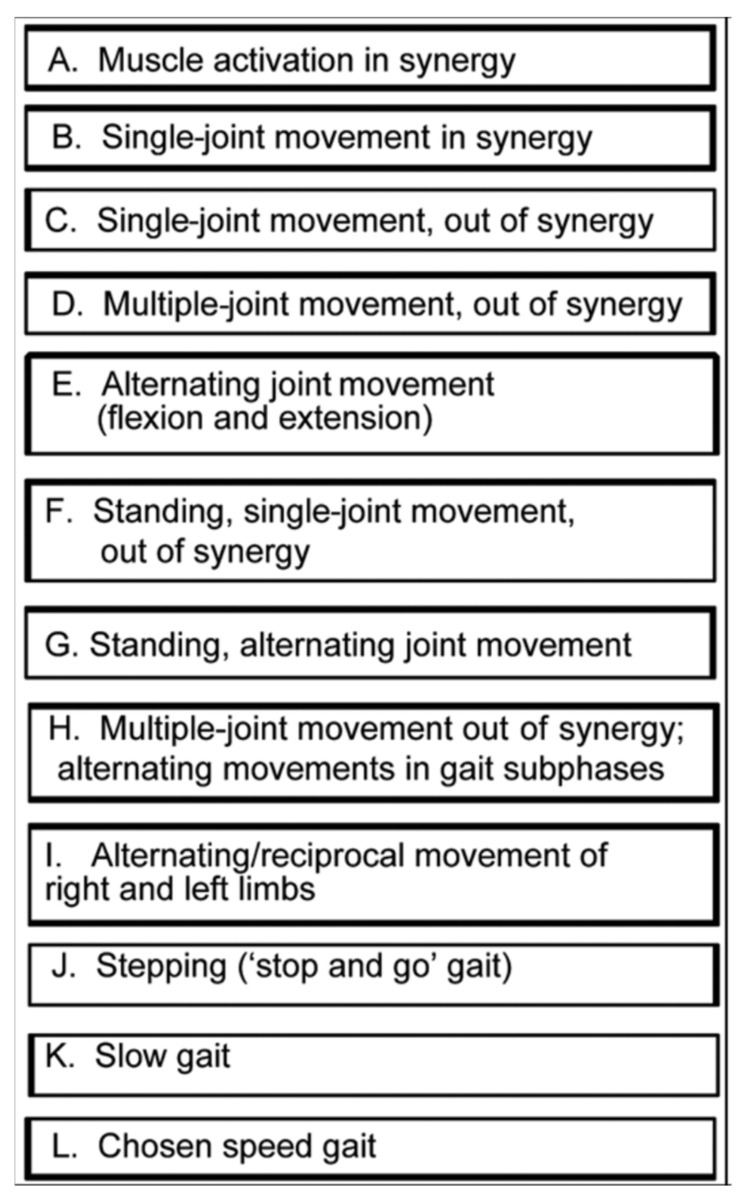
Motor Task Hierarchy for Limb and Gait Coordination Training (with permission, JRRD, 2012, Daly et al. [31]).

At the initial treatment session, assessment was conducted and exercises commenced at the custom level for this participant. Factors contributing to both the initial level of assigned exercises and progression of the program included the following: presence of coordination impairment (limited active joint movement tested in an array of body positions and activities); speed of movement limitations (Figure 1); baseline strength; gait coordination deficits and compensatory strategies employed. For the hip, knee, and ankle movements that were impaired and relevant to gait swing phase, we also assessed the ability to volitionally move along with assist devices such as functional electrical stimulation (FES, parallel bars, walker, and cane). For each lower limb movement required in swing phase, we followed the hierarchy in Figure 1 using the following motor ability characteristics in order to assign the exercises:

1. “Percentage of the normal range of movement that could be executed, volitionally and independently.
2. Percentage of the motor task that could be executed with the support of verbal or tactile facilitation.
3. Percentage of the normal range of movement that could be executed along with an assistive movement device.
4. Normality of effort level during the task (e.g., holding breath, abnormal co-contraction of muscles distant from the targeted task joints or antagonist muscle contractions).
5. Compensatory strategies employed during execution of the motor task.
6. Percentage of the task for which compensatory strategies were employed.
7. Number of repetitions of the motor task that could be performed with only a “beat” between repetitions before the motor task was performed in an “abnormal fashion” (with permission [31]).

After the initial assessment, motor learning commenced to improve hip, knee, and ankle joint movement coordination required during gait swing phase. The motor learning protocol was based on well-known principles of both brain plasticity and learning, including the following: motor task-specific practice, which entailed practice as close-to-normal joint movement coordination as possible [32,33,34,35] with continual progression toward normal, as ability improved; high repetition of the desired coordinated joint movements [36,37,38]; attention focused on the motor task at hand [39], training specificity [40,41,42], which in this case entailed practice of gait swing phase movement components; and awareness and feedback [43] regarding the normal desired movement compared to the abnormal characteristics of the movements performed [31].

#### 2.3.2. FES Assist in Motor Learning

It is well-known that repetition of abnormal movements is counterproductive in coordination training. Therefore, FES was used as a practice-assist for knee and ankle movement practice. With FES-assist, Mr. X was able to perform more coordinated movements during practice. In this way, FES-assisted movement also allowed Mr. X to practice a given movement task earlier in the program than would have been possible using his volitional effort alone, and he was able to replicate this movement more consistently during home practice with the assist of FES [44,45,46].

Because this participant was a part of a larger study [47], the FES was provided via electrodes implanted beneath the skin, under anesthesia to ensure complete comfort of the participant. He received a system that could activate knee flexors, knee extensors, and ankle dorsiflexors and evertors. The electrodes were constructed of very fine material that was not perceived by the participant after placement. When study participation was complete, the electrodes were removed. There were no adverse events in relationship to the FES procedures. FES signal was activated via a finger switch by the participant during exercise practice and by the therapist during gait training practice. FES parameters were as follows: 30 Hz, 20 mv amplitude, and pulse width customized according to complete comfort of the participant. The duty cycle could be set for on/off timings for up to 20 s. The custom FES stimulator allowed for the physical therapist to generate gait swing phase FES-assist patterns for knee and ankle muscles which were timed according to the needs of the participant as he progressed in volitional ability. As volitional ability improved, FES levels were progressively reduced. We assigned home practice of the exercises, performed in-person, including FES-assisted movements.

#### 2.3.3. Progression from Side-Lying to Sitting Positions and Upright Standing

Rules for treatment progression. We progressed treatment according to following rules for a given volitional joint movement [31]:

1. Fifty percent of normal range of movement is executed, volitionally, independently; or 50 percent of motor task is executed with support of verbal or tactile facilitation; or 50 percent of normal range of movement is executed, along with motor assist device.
2. Normal level of effort is expended during task (no holding breath or associative reactions in other limbs or trunk; relaxed uninvolved muscles).
3. If motor compensatory strategies are employed, less than 10 degrees of movement is performed that is compensatory in nature.
4. If motor compensatory strategies are employed, at least half of motor task is performed without compensatory strategies.
5. Five or more repetitions of motor task can be performed in a row with only a “beat” between before motor task deteriorates into uncoordinated or incorrect.

##### Body Position and Limb Position Progression

Side-lying position

**Figure 2:**
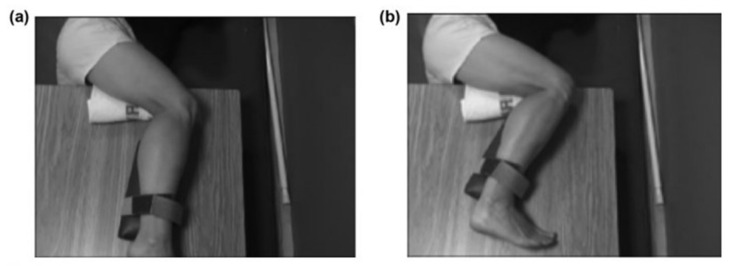
In-Synergy Position for Knee Flexion Practice. Panel (**a**) is the start position of the exercise. The position is shown from a semi-aerial view, looking down on the exercise board (powder board with short wooden legs to hold the board off the mat and off the uninvolved leg, which is resting on the mat). The position of training is side-lying with the uninvolved leg resting on the mat. The involved leg is resting on the top of the exercise board. The in-synergy start position for knee flexion practice is with the joint(s) contiguous to the knee (hip and ankle) in a flexed position. Panel (**b**) shows the end position of the knee flexion exercise, with the knee as fully flexed as can be performed. The hip is in a flexed position throughout (with permission, JRRD, 2012, Daly et al. [31]).

**Figure 3:**
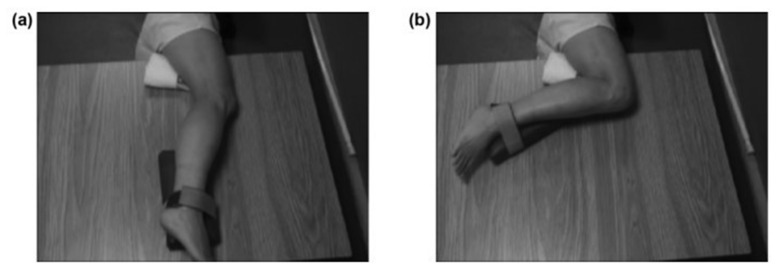
Out of Synergy Position for Knee Flexion Practice. Panel (**a**) is the start position for the out-of-synergy position for knee flexion practice. The out-of-synergy exercise is more difficult in that the hip is no longer in a flexed position as in Figure 2. Rather, the hip is in an extended position or neutral position, as shown here, at the start of the exercise, and the participant is directed to maintain this hip position throughout. Panel (**b**) shows the end position of the motor task, which is to flex the knee and maintain the hip in extended or neutral position (with permission, JRRD, 2012, Daly et al. [31]).

For recovery of motor control after stroke, task practice assignment most productively includes consideration of not only the factors listed above, but also, the body position with respect to gravity during the exercise. The initial body position was selected according to coordination capability and progressed according to the above rules. We observed that knee extensor abnormal co-contraction was lessened in the side-lying position with the right stroke-involved limb uppermost. We positioned a ‘powder-board, exercise board under the right lower limb, so that the hip, knee and ankle could be extended and flexed in the horizontal plane with ‘gravity-eliminated’. Initially, Mr. X practiced knee flexion in this side-lying position, with the hip maintained in a static and flexed position (Figure 2). This was the only whole limb position in which he could produce even partial knee flexion movement under his own volition. As his coordination began to return, he was able to partially flex the knee with the static hip position progressively and gradually lessened in its hip-flexed position and moved to a hip-neutral position (Figure 3), and then to a hip-extended position. We continued the challenge level by subsequently assigning practice of knee flexion during the dynamic paradigm of simultaneous hip extension movement; this created an exercise of extension movement at the hip and flexion movement at the knee, which is considered an ‘out-of-synergy’ exercise (Figure 1, row D).

Sitting and standing positions

Using the progression rules above, we progressed him first to a sitting and then to a standing position for practice of knee flexion. The sitting practice included knee flexion practice that was progressed through greater range of movement, as he was able to volitionally perform the movements without evidence of compensatory movements. The first standing position was with body weight supported on the left limb (parallel bars, hand hold) and the right hip in minimal flexion and the right toe resting on the floor which created some knee flexion, as the starting position. We progressed the standing exercise to a start position with the right limb fully extended at the knee with the right foot resting lightly on the floor.

Awareness training

We provided awareness training throughout the interventions so that Mr. X learned to identify and differentiate between coordinated movement versus dyscoordinated movement [43].

#### 2.3.4. Body Weight Support

Body weight support was used at the point in Table 1 hierarchy when Mr. X progressed to coordination training in the upright standing position (Table 1, row F). We titrated weight support to vary the level of pressure on the plantar surface of the stroke-involved limb. For some stroke survivors, excessive extensor muscle activation is abnormally activated apparently in response to greater joint and plantar-flexor surface pressure that occurs in the upright standing position or during walking [9,10,13]. Initially, we used the unweighting feature of the harness system to lessen the pressure on the right limb joints and plantar surface at pre-swing. Subsequently, as the participant gained volitional control to mitigate extensor muscle activations of hip, knee, and ankle, we progressively reduced the support, effectively increasing the forces on the right limb joints and plantar surface during practice. One difficulty that a stroke survivor experiences is flexing the involved knee immediately after the weight bearing that occurs during stance phase. Therefore, it was important to practice the coordination problem inherent in transitioning from activating the involved limb knee extensor muscles during weight bearing to immediately activating the knee flexor muscles required during swing phase. Exercise progressions are provided in the following example, for the involved knee flexion required at initial swing, just subsequent to involved limb weight bearing:

Exercise 1. Start position of unweighted body, static position, knee flexion exercise: using about 30% weight support-assist, stand in the stride position with the uninvolved limb in front and bearing all the remaining body weight; place the involved limb in the trailing position with the toe touching the ground, bearing no weight. Flex the knee volitionally. If there is very little active movement, FES with surface electrodes can be used to assist in activating knee flexors. With mastery of knee flexion in this position, and with no compensatory movements, progress to exercise 2.

Exercise 2. Start position, as above for Exercise 1, except that a small amount of weight is borne by the involved toe, touching the ground in the toe-touch, trailing limb position. Translate body weight fully forward to the uninvolved limb mid-foot and hold that single-leg standing position, effectively unweighting the involved limb. Flex the involved knee. Exercise 2 is more difficult than Exercise 1, due to the addition of a small percent of weight bearing borne at the beginning of the exercise on that involved toe, in the toe-touch position.

Exercise 3. Start position, as above, but now progressively increase weight borne in the start position by the involved limb forefoot. Progress the difficulty by beginning the exercise with weight borne on the entire plantar-flexor surface of the trailing involved limb.

Exercise 4. Once normally coordinated knee flexion can be achieved using the weight support system, gradually decrease the level of weight support until the individual can perform the knee flexion of initial swing phase that should occur immediately after involved limb stance phase, bearing full body weight.

Exercise 5. Begin more rapid transfer of weight from involved limb followed by subsequent knee flexion. With mastery of more rapid transition from weight bearing to knee flexion, the following exercises can be initiated.

Exercise 6. Begin stepping practice on the treadmill or overground with about 20–30% weight support, with slow walking or at the slowest treadmill speed (e.g., 0.2 m/s), to ensure that knee flexion at initial swing is performed without compensatory movements. FES-assist can be used at initial swing for this slow practice.

Use high repetition practice, allowing the participant to manage more and more body weight, as volitional swing phase flexion improves.

Repeat above until full swing phase limb flexion is occurring at the correct gait event time of mid-swing, and full body weight is managed by the participant.

Much of the feedback was initially extrinsic (provided by the training therapist). However, very early in the program, the individual participated in awareness training, learning to self-assessed performance, relying then on intrinsic feedback.

#### 2.3.5. Treadmill Use

In addition, the body weight support system was combined with a treadmill. This combination was used to progressively train speed of movement of the involved limb during swing phase limb flexion. Initially, the treadmill speed was at the lowest possible setting, at 0.2 m/s or less. As swing phase coordination improved, treadmill speed was progressively increased. Additionally, we titrated body weight support to enable faster swing phase practice than would have been possible initially. Initially FES was also incorporated to cue knee flexion; activation of the knee flexion stimulus was controlled by the therapist for each practiced step. As volitional control improved, the level of FES was reduced concomitantly.

### 2.4. Data Analysis

For this case report, descriptive data were generated for all measures at pre- and post-treatment. From the 6MWT measure, we derived speed of walking.

## 3. Results

Participant baseline measures are listed in Table 1. Swing phase gait deficits at baseline were as follows: absent right hip and knee flexion during mid-swing phase of the right, involved limb (“stiff-legged gait”), and ankle inversion/plantarflexion during swing phase. Compensatory strategies included slow gait speed and the following compensatory strategies during right swing phase: short stride length (sliding the right foot across the floor, to a ‘step-to’ position next to the uninvolved stance limb); left leaning during left stance phase. Observation of the gait pattern during right pre-swing revealed that he achieved less than normal knee flexion at right pre-swing, but then he subsequently proceeded to abnormally extend the knee throughout early and mid-swing phase, rather than flexing the knee to peak swing flexion of 60°, which would have been the normal swing phase limb progression to mid-swing. We tested knee flexion coordination in the static body position, first in a sitting position, with the right heel on the floor out in front of the body; Mr. X was unable to slide the heel toward the body by flexing the knee (0 degrees of movement). Second, in a standing position on the left foot (hand support in parallel bars), with the right toe resting on the floor behind the body, Mr. X was unable to actively produce flexion of the knee. Third, standing on the left foot with the right plantar surface resting on the floor, Mr. X was unable to produce active knee flexion (0 degrees of knee flexion).

**Table 1:** Results for Impairment, Mobility Function, and Life Role Participation Measures.

A. Measure	B. Pre	C. Post	D. Gain/Improvement
**Impairment Measures**			
1. FM Total Score (34, max); Items that changed:	27	30	3
Item: Ankle dorsiflexion,	1	2	1
Item: Knee Flx, sit, combined synergy	3	4	1
Item: KnFx, Stnd, out of synergy	2	3	1
2. mAshworth (0, normal), item that changed:			
Item: Knee extensors	1	0	1
3. Muscle Strength			
External hip rotators	4+	5	gain
Internal hip rotators	3−	3−	No change
Knee flexors	4	4	No change
Ankle evertors	4+	4+	No change
All other muscles	5	5	No change
4. G.A.I.T. Overall Score (0 is normal; item scores, Table 2)	25	13	12
**Functional Mobility Measure**			
5. 6 MWT (feet)	582	1114	532 *
meters	77	339	162
Speed (derived from 6MWT)	0.49 m/s	0.94 m/s	0.45 m/s
	* MCID > 233′ [29].		
**Life Role Participation Measure**			
6. SIS (Domain scores in separate Table below)	293	305	12 *
	* MCID = 10–15 points [30].		

### 3.1. Impairment

Muscle strength. There was no change in strength from pre- to post-treatment for most muscle groups. Strength for all hip muscle groups ranged 4 to 5, except for hip rotators. Internal rotators showed a grade of 3− throughout, and hip external rotators improved from 4+ to 5 at post-treatment. Knee flexors were a grade of 4 throughout, and knee extensors were a grade of 5 throughout. Ankle plantar-flexors, dorsiflexors, and invertors were grade 5 throughout. Ankle evertors were a grade 4+ throughout (Table 1, item 3).

mAshworth. At baseline, mAshworth measured 0 (normal) for hip flexors, extensors, abductors, adductors, and external rotators. Hip Internal rotators were a score of ‘1′ throughout. Also, normal throughout were knee flexors and ankle dorsiflexors. Ankle plantar-flexors scored a ‘1′ throughout. Knee extensor baseline score of 1 improved to normal (0) by post-treatment (Table 1, item 2).

Limb Coordination. The FM coordination scale showed improvement in three items (listed in Table 1, item 1).

Gait Coordination. The G.A.I.T. showed improvement of 12 points (Table 1, item 4; lower score is an improvement). Table 2 lists each of the items which showed improvement. Seven items showed improvement to normal (normal = 0), and one item showed a one-point improvement.

Swing phase Kinematics

**Figure 4:**
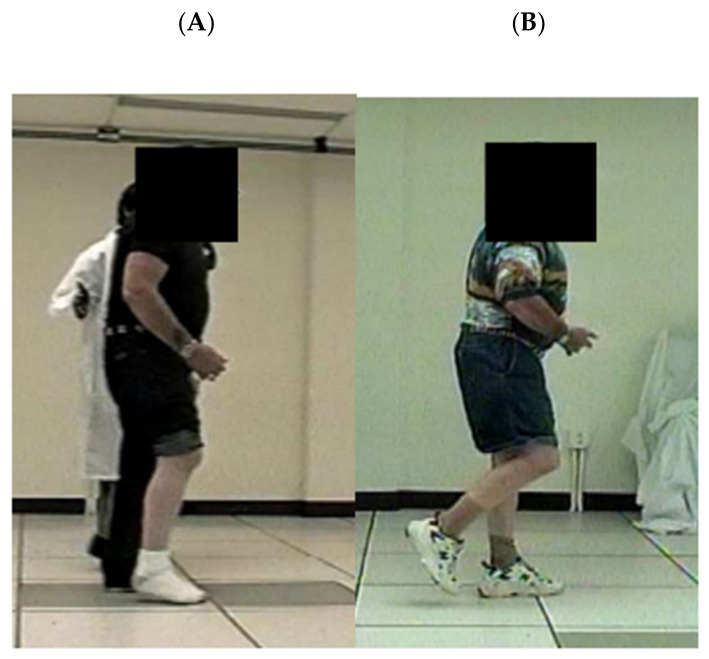
Change in Swing Phase Hip, Knee, and Ankle Flexion After Treatment. Panel (**A**). Pre-Treatment, Mid-Swing Gait Dyscoordination. At mid-swing, hip and knee flexion are absent, and the ankle is slightly plantar-flexed. The right plantar surface is sliding across the floor in order to bring the right limb at least up to the left stance limb. Panel (**B**). Post-Treatment, Mid-Swing Improved Hip, Knee, and Ankle Flexion. At mid-swing, the right hip, knee, and ankle are flexed, which is necessary in order for the right limb to clear the floor during swing phase (with permission, Stroke 2006, Daly et al. [47]).

Mid swing hip flexion improved 25° (Table 3 row 1, column D). Knee flexion at toe off improved 12.0° (row 2). Mid swing knee flexion improved 32° (row 3). Ankle dorsiflexion at mid-swing improved 13°, from 7° in the plantarflexion range to 6° of dorsiflexion. Further insight into the baseline swing phase deficit can be obtained by inspecting Table 3 at pre-treatment (column B). That is, there was less than normal knee flexion at toe-off (23°; row 2), with subsequent further abnormal extension at the knee while progressing to mid-swing (row 3), at which time, the knee was in even less knee flexion at 12° (row 3), when the normal progression should have been increasingly greater flexion of the knee from toe-off to mid-swing (column E, rows 2 and 3, respectively). At baseline, Figure 4A illustrates the gait event just before mid-swing for one stride; at this point; the involved swing phase limb has been moved forward with the thigh just anterior to the uninvolved stance limb thigh. From this photograph, baseline gait deficits are apparent as follows: markedly reduced hip, knee, and ankle flexion, as well as abnormal plantarflexion (Figure 4A). In contrast, at post-treatment (Figure 4B), there was recovery of some knee flexion and hip flexion, as well as ankle dorsiflexion. Notably at post-treatment, we observed that there was an absence of the baseline compensatory strategies of left stance phase leaning, shortened swing phase, and sliding of plantar surface along the floor during swing phase.

Muscle activation latencies

There was a pattern of change from pre-treatment to post-treatment during the two static body knee flexion tasks (side-lying and standing; Supplementary Table S1). Pre-treatment latencies ranged from 450–650 ms (column B), whereas post-treatment latencies ranged from 210–320 ms (column C). By comparison, healthy adult latencies during these two knee flexion tasks ranged from 200–265 ms (LH and VM; [28]).

Results of muscle latencies during walking are shown in the Supplementary Figures S1–S4. Hamstrings latencies improved to close to normal for both stance and swing phase (Figure S2). Quadriceps latencies improved during swing phase, showing the normal pattern of no activation during initial swing (at 60% of the gait cycle, beginning of swing phase) and normal onset of activation at 99% of the gait cycle in late swing to extend the knee in preparation for the subsequent heel strike (Figure S3). The Gluteus Maximus (hip extension) showed improvement of no abnormal activation during swing phase from 60–95% of the phase (Figure S4).

### 3.2. Functional Mobility

There was improvement in the 6MWT of 532′ (Table 1), which is clinically significant (MCID > 233′ [29]). We can note that the gait speed derived from the 6MWT showed improvement from 0.49 m/s to 0.94 m/s. Considering this change within Perry’s velocity-based ambulation classifications, there was an improvement from the lowest value of ‘limited community ambulator’ (0.4–0.8 m/s) to a full community ambulator (>0.8 m/s [49]).

### 3.3. Life Role Participation

Table 1 shows an overall SIS improvement of 12 points, which is within the threshold of a clinically meaningful change [30]. Table 4 lists the separate domains of the SIS and shows that there was improvement in the following domains: (1) physical/mental problems due to stroke; (5) daily activities; (6) mobility; (7) hand function; and (8) meaningful activities.

## 4. Discussion

This case study contributes to the literature in several ways: (1) illustrates that strength is necessary, but not sufficient to restore coordinated gait swing phase after stroke in some stroke survivors; (2) provides supporting case evidence of relationships among knee flexion coordination, swing phase coordination, and functional mobility; (3) illustrates combined interventions for gait training and response to treatment; and (4) provides details regarding coordination training and progression of gait training treatment for stroke survivors.

### 4.1. Strength Is a Necessary Pre-Requisite, but Not Sufficient to Restore Normal Gait after Stroke, if Gait Dyscoordination Is Present

A number of researchers have published work that describes the necessity of strength for mobility. For example, others have, reported relationships between strength improvement and gait speed [17,18,19,20], walking endurance [15,16,20], and balance and functional capability [21], but there is mixed evidence regarding recovery of function as a result of strength training. For example, Dorsch et al. [50] conducted a systematic review of results from progressive resistance training; they reported a large effect size regarding strength improvement, but notably, there was no definitive effect at the level of activity. Further, many studies have added importantly to knowledge in the field regarding possible gait training interventions but did not report recovery to normal for gait coordination, function, and life role participation (e.g., [15,16,17,18,19,20,21,51]). This situation begs the question, “What is the missing critical ingredient(s) in gait training interventions which would be sufficient to consistently produce functional and quality of life changes?”. It is reasonable to consider that one of the critical ingredients is coordination training, of both the lower limb and the dynamic situation of gait. To support that possibility, we can note that others have reported a relationship between abnormal coordination and gait speed (e.g., [52,53,54]); and some studies have also reported that coordination training can improve walking speed (e.g., [46,52]) and gait coordination (e.g., [46,55]). This current case report is informative because it highlights response to coordination training for a stroke survivor who had normal or close to normal muscle strength at the baseline of the treatment protocol. The response to treatment for this individual is informative in a manner not always possible, because muscle weakness was not present at baseline, muscle strength remained constant throughout, coordination impairment was present at baseline, and coordination of lower limb and gait improved. These improvements in gait coordination were observed with concomitant improvements in measures of function and life role participation, highlighting that, even when strength is nearly normal, precisely targeted training of coordination is required to restore more normal movement control. This case report provides an example of highly customized training that incorporated a multi-modal, patient-centered treatment plan.

### 4.2. Relationship between Knee Flexion Coordination and Swing Phase Gait Coordination

‘Out-of-synergy’ motor tasks prove difficult for many stroke survivors because of persistent abnormal co-contractions [9,10] of the lower limb muscles. In fact, gait dyscoordination after stroke can entail abnormal timing of muscle activations that create conflicting co-contractions of agonist and antagonist at both ankle and knee [8]. Normally the coordinated knee flexion movements associated with swing phase progress from 0° to 35° from pre-swing to toe-off, and then to a peak flexion value of up to 60° at about 80% of the gait cycle during swing phase [48,56]. At baseline, Mr. X’s swing phase pattern was reversed, with peak knee flexion occurring too early at toe off, followed by the abnormal movement of knee extension by mid-swing (Figure 4A). This baseline dyscoordination pattern is illustrative of abnormal muscle co-contractions during walking [9,10].

For Mr. X, there was improvement in both limb coordination and in gait swing phase flexion of hip, knee, and ankle. The FM lower limb coordination scale showed improvement in two items pertaining to knee flexion limb coordination. First, there was a return to normal for knee flexion coordination performed in the seated position, which is considered a ‘combined synergy’ motor task because all three joints of the lower limb (hip, knee, and ankle) are either stationary and in a flexed position throughout the movement or are moved into flexion during the motor task. Second, there was improvement for knee flexion performed in the standing position, which is considered an ‘out-of-synergy’ motor task. This is considered ‘out-of-synergy’ because the task is knee flexion, but the contiguous joint, the hip joint, is not in a flexed position (that is, the hip is maintained in a ‘neutral’ position during the knee flexion movement). Finally, subsequent to improvement in stationary knee flexion motor tasks, we observed emerging ability to flex the lower limb during the standing exercise progression described in Methods. Ultimately, with continued practice, Mr. X was able to gain control of limb flexion during the dynamic situation of walking. At post-treatment, the limb flexion pattern followed the normal progression of greater and greater flexion until mid-swing, allowing this stroke survivor to discard prior compensatory strategies. In stroke survivors with impaired knee flexion coordination during the side-lying task described in the current work, correlations were previously reported between impaired muscle activation (onset times for knee flexors (LH)) versus joint limb dyscoordination (FM; r = 0.73) and versus walking distance/speed (6MWT; r = 0.62; [28]). Additionally, for the standing knee flexion task described in the current work, correlations were previously reported between impaired muscle activation (onset times for knee flexors (LH)) versus joint limb dyscoordination (FM; r = 0.62) and versus walking distance/speed (6MWT; r = 0.56; [28]). Given the existing evidence from others, prior work, and the timing and nature of motor control improvement in this case, it is reasonable to consider that the improved swing phase limb joint coordination was associated with the critical and improved limb flexion coordination measured by the FM lower limb coordination scale.

### 4.3. Relationships among the Variables of Gait Coordination, Gait Speed, Function, and Life Role Participation

Many have studied potential relationships among gait coordination, gait speed, function, and life role participation [46,52,53,54,55]. Gait speed and life role participation appear to be correlated [57,58]. Strong evidence exists regarding relationships among functional abilities and quality of life (e.g., [59,60,61]). In this case report, we demonstrated that a targeted intervention focused on swing phase coordination produced measurable change not only in measures of gait coordination, but also in functional mobility and life role participation. It is possible that since the training protocol was specifically customized to target the array of his deficits, a robust change across measurement domains was realized. Treatment of impaired gait speed and endurance are frequently emphasized with the stroke population. This case report suggests that addressing the underlying limb and gait coordination impairment can also beneficially impact patient outcomes [9].

### 4.4. Advantages of the Combined Interventions

It is well-known that stroke can cause different combinations of numerous impairments, with each impairment exhibiting along a separate continuum of severity. Examples of impairments relevant to gait deficits after stroke include abnormal muscle co-contractions and inability to perform coordinated movements of the lower limb and gait pattern [7,8,9,10]; muscle atrophy, weakness, and tissue change [62,63]; imbalance [6]; and poor walking endurance [15,16,20]. Because of this broad variety of symptomatology and severity, it is important to apply ‘precision medicine’ (precision neurorehabilitation) when evaluating and customizing treatment. The literature is replete with examples of useful interventions that reported promising improvements for one type of impairment, but with less or no recovery in other impairments and lack of recovery in function and life role participation. In the presence of multiple impairments, it is obvious that the most optimal treatment plan will address all the impairments precluding recovery of function. In the situation of persistent gait dyscoordination, it is imperative to employ methods that address the specific dyscoordinated movements in the gait pattern. It is logical then to incorporate a combination of the most effective known types of coordination and gait interventions that best target the impairments underlying gait dyscoordination.

In this case report, first, motor learning was applied, as described in the Methods section, in a manner based on well-known basic research evidence and targeted to produce brain plasticity for recovery of coordination [32,33,34,35,36,37,38,39,40,41,42]. Second, FES was employed as a practice-assist device to guide perception and coordinated muscle contractions. We applied FES also according to known motor learning principles. For example, FES-assist enabled practice of more normally coordinated movement and higher repetition practice [64] both in the clinic and during home exercise. The result was that FES-practice assist was advantageous in that more complex coordination could be practiced productively versus with volitional effort alone. That is, more normal isolated joint movements could be practiced, if FES was applied in conjunction with volitional exercise [46,55]. Studies have reported that FES has proven beneficial according to the following measures: gait speed, balance, spasticity and ROM [65]; strength and balance [66]; ankle control and gait parameters [67]; ankle dorsiflexor and plantar flexor gait characteristics [68]; and improvement in gait coordination maintained at least 6 months after the last treatment [46]. In the current case report, the implanted electrode system was advantageous compared to surface FES systems because the stimulating electrodes were beneath the skin at the ‘motor point’ of each respective muscle. Because the skin pain receptors were not activated, it was possible for the participant to request higher and comfortable stimulation levels for a given muscle, to advance coordination practice. Additionally, the multi-channel FES system and custom stimulator allowed for the knowledgeable neurorehabilitation therapist to construct FES-assist swing phase gait patterns that were customized not only to this participant, but also progressed daily, as needed, in order to precisely assist and challenge the gait component practice for a particular day. But at the same time, surface FES could be used effectively in a similar fashion and inherently is a more practical, accessible, and non-invasive method for using FES-assist in coordination training (e.g., [69]). For practice of joint movement coordination, multi-channel, table-top FES equipment can be obtained. For gait practice, FES stimulators with foot switches or finger switches could work well. There is a need for FES stimulators that can be more versatile in stimulus activation methods and timing flexibility [70,71].

Third, we employed weight-supported training in the upright, standing position and during very slow gait component practice. Fourth, we used overground gait training and treadmill training (along with weight support). A systematic review of treadmill training in subacute and chronic stroke survivors (974 subjects; [72]) found that treadmill training had no effect on walking independence, but did increase walking speed and endurance (n.b., subacute patients could have had spontaneous improvement). This same review found that body weight supported treadmill training had no benefit beyond that of treadmill training without weight support [72]. There was not enough information in the studies reviewed to identify any useful functional or quality of life improvement with either modality. In the current study, neither treadmill training nor weight support were used in a formulaic manner; rather, these two modalities were incorporated within a motor learning program and they were used as described in the methods examples to finely incrementalize certain exercises in the hierarchy of motor learning challenges assigned for exercise practice. Advantages of weight support included the following: lessening pressure on the plantar surface of the foot and through the stance phase limb joints; mitigating fear of falling in the standing position with weight borne on one leg for limb flexion practice; providing a safe practice environment for weight shifting from the trailing limb to the forward limb; and availability of a continuum of weight support for incremental challenge. Advantages of treadmill training (with weight support) included practice of progressively faster transition from stance to swing and from toe-off to mid-swing. Considering the use of each tool used in the coordination training for this case report, one can appreciate that the array of tools, taken together, offered numerous possibilities for productively challenging and progressing limb coordination and gait coordination training.

### 4.5. Limitations

This work has the standard limitations of a case report, in terms of generalization to the stroke population. At the same time, the case evidence reported here is relevant to the rehabilitation of other stroke survivors who present with the simultaneous factors of normal strength needed for walking, but swing phase coordination deficits. This case evidence also supports the importance of further study of the treatment response to coordination training for stroke survivors.

## 5. Conclusions/Contribution to the Field

This case report illustrates, for gait dyscoordination, the advantages of combined interventions within a motor learning paradigm and provides details regarding coordination training and progression of gait training treatment for stroke survivors. The response to treatment provides supporting case evidence of relationships among knee flexion coordination, swing phase coordination, functional mobility, and quality of life. Because of this case’s unique situation of normal or close-to-normal baseline strength, but abnormal gait coordination, case results illustrate that, although strength is necessary, it was not sufficient in this case to restore coordinated gait swing phase after stroke in the presence of persistent gait dyscoordination.

## Figures and Tables

**Table 2:** Gait Assessment and Intervention Tool (G.A.I.T.), Measuring Gait Coordination.

G.A.I.T. ITEMS Showing a Gain	Pre	Post	Gain Score
**Stance phase, item number**			
6, trunk lat flx	1	0	1
7, trunk wgt shift	3	0	3
12, knee loading	1	0	1
13, knee flx midstance	2	1	1
15, ankle plntarflx	1	0	1
17, ankle plntarflx, term pre-swing	2	0	2
**Swing Phase, Item number**			
23, knee initial swing	1	0	1
26, ankle sagtl	1	0	1
27, ankle inversion	1	0	1
**Gain score for items that changed**			12

**Table 3:** Gait Kinematic Improvements (degrees of motion).

A. Joint Movement and Gait Event	B. Pre-Treatment	C. Post-Treatment	D. Gain	E. Normal *
1. Mid-Swing Hip Flexion	9	34	25	35
	(±3)	(±2)		(±3)
2. Knee Flexion at Toe-Off	23	35	12	35
	(±3)	(±3)		(±5)
3. Mid Swing Knee Flexion	12	44	32	60
	(±3)	(±2)		(±4)
4. Mid Swing Ankle Dorsiflexion	−7	6	13	4
	(±3)	(±2)		(±4)

* Neuman 2013 [48].

**Table 4:** SIS Domains.

Domain	Pre-Treatment	Post-Treatment	Change Score
1. Physical/mental problems due to stroke (x/20)	15	17	2
2. Memory and thinking (x/40)	40	40	0
3. Mood/emotions post stroke (x/45)	40	39	−1
4. Communication and understg. (x/35)	35	35	0
5. Daily activities (x/60)	55	59	4
6. Mobility (x/50)	46	49	3
7. Hand function (x/25)	20	23	3
8. Meaningful activities (x/45)	42	43	1
Total	293	305	12

## Data Availability

This is a case report. All the data associated with this paper are provided in the manuscript and Supplementary Files.

## Supplementary Materials

The following are available online at https://www.mdpi.com/article/10.3390/brainsci11111498/s1, Figure S1 Tibialis Anterior (Ankle Dorsiflexor) Muscle Activation Latencies During Walking at Chose Speed; Figure S2 Hamstrings, Figure S3 Quadriceps, and Figure S4 Gluteus Maximus Muscle Activation Latencies During Walking at Chose Speed; and Table S1: Muscle Activation Latencies During Two Static Body Knee Flexion Motor Tasks.
