# Supplementary material for: Necessity and Content of Swing Phase Gait Coordination Training Post Stroke; A Case Report
Source: Brain Sci. 2021 Nov 12;11(11):1498. doi: 10.3390/brainsci11111498 (PMC8615654; doi:10.3390/brainsci11111498)
Supplement: Supplementary file 1 [file brainsci-11-01498-s001.zip › brainsci-1420640-supplementary.pdf]

SUPPLEMENTARY FILE OF TABLE S1 AND FIGURES S1, S2, S3, AND S4

Table S1. Muscle Activation Latencies During Two Static Body Knee Flexion Motor Tasks

A. Task and Muscles	B. Pre (ms)	C. Post (ms)
1. Sidelying Knee Flexion with Hip in Neutral Static Position		
Knee Flexors		
Short Head Biceps Femoris	520 (± 200)	250 (±130)
Long Head Biceps Femoris	520 (±190)	220 (±90)
Knee Extensors		
Vastus Medialis	540 (±190)	300 (±160)
Vastus Lateralis	560 (±170)	320 (±120)
2. Standing Knee Flexion with Right Limb in Trailing Limb Position and Toe Touching the Ground		
Knee Flexors		
Short Head Biceps Femoris	510 (±80)	250 (±120)
Long Head Biceps Femoris	450 (±82)	210 (±140)
Knee Extensors		
Vastus Medialis	650 (±150)	280 (±150)
Vastus Lateralis	648 (±140)	300 (±130)

Figure S1. Tibialis Anterior (Ankle Dorsiflexor) Muscle Activation Latencies
During Walking at Chose Speed

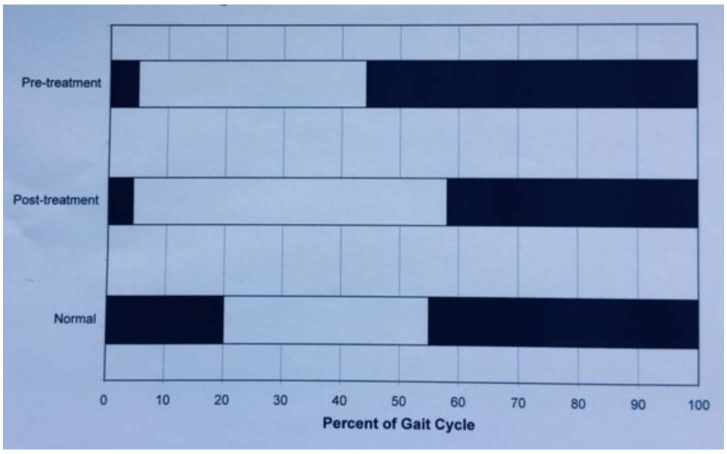

Figure S1. shows improvement toward normal (bottom panel) from Pre (top panel) to Post-treatment (middle panel) at the beginning of swing phase (60%)

Figure S2. Hamstrings (Long Head, Biceps Femoris, Knee Flexors) Muscle Activation Latencies
During Walking at Chose Speed

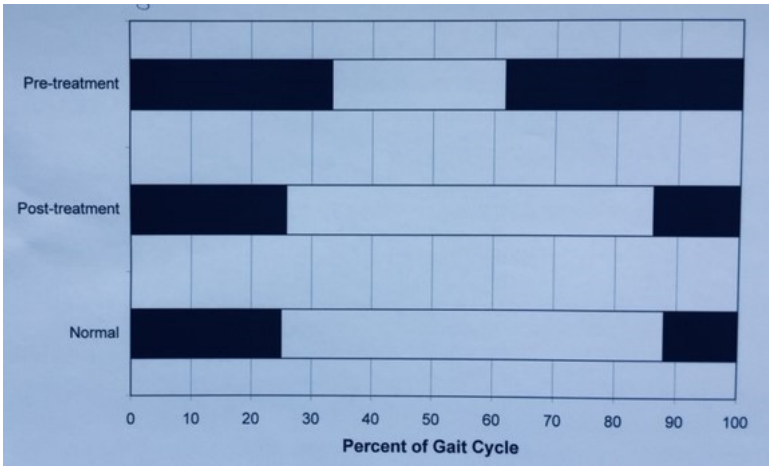

Figure S2. shows improvement toward normal (bottom panel) from Pre (top panel) to Post-treatment (middle panel) during stance phase and during swing phase (60 - 100%).

Figure S3. Quadriceps (Knee Extensors) Muscle Activation Latencies During Walking at Chose Speed
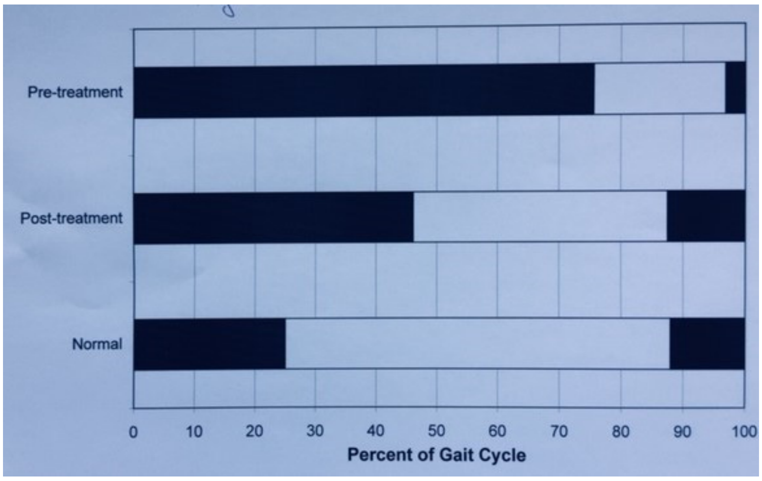
Figure S3. shows improvement toward normal (bottom panel) from Pre (top panel) to Post-treatment (middle panel) during stance phase and during swing phase (60 - 100%).

Figure S4. Gluteus Maximus (Hip Extensors) Muscle Activation Latencies During Walking at Chose Speed
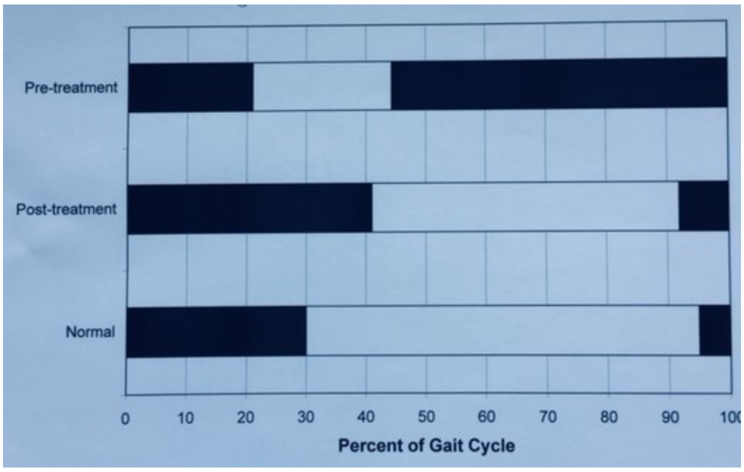
Figure S4. shows improvement toward normal (bottom panel) from Pre (top panel) to Post-treatment (middle panel) during stance phase and during swing phase (60 - 100%).
